# Status of metals in serum and urine samples of chronic kidney disease patients in a rural area of Bangladesh: An observational study

**DOI:** 10.1016/j.heliyon.2021.e08382

**Published:** 2021-11-12

**Authors:** Tasrina Rabia Choudhury, Sk. Zubaer Zaman, Tanzina Iveen Chowdhury, Bilkis Ara Begum, Md. Anwarul Islam, Md. Mostafizur Rahman

**Affiliations:** a Analytical Chemistry Laboratory, Chemistry Division, Atomic Energy Centre Dhaka, Bangladesh Atomic Energy Commission, Dhaka, 1000, Bangladesh; b Department of Chemistry, University of Dhaka, Dhaka, 1000, Bangladesh; c Department of Obstetrics and Gynaecology, Bangabondhu Sheikh Mujib Medical University (BSMMU), Dhaka, 1000, Bangladesh; d Department of Environmental Sciences, Jahangirnagar University, Dhaka, 1342, Bangladesh

**Keywords:** CKD, Copper, Zinc, Urine, Cadmium, Chromium, Lead

## Abstract

The traditional causes of Chronic Kidney Damage (CKD) are Diabetes and Hypertension. However, recent studies reported the possible relations between metal exposure and CKD. This study aims to explore the status of metals in CKD patients compared to their healthy counterparts at Narayanganj, Bangladesh, through a cross-sectional study. In this study, 50 volunteers were involved; 30 CKD patients and 20 healthy controls. Five metals were measured from serum [Copper (Cu) and Zinc (Zn)] and urine [Lead (Pb), Cadmium (Cd), and Chromium (Cr)] using Atomic Absorption Spectrometry. Compared to the controls the CKD patients exhibited significantly higher levels of Pb, Cd and Cr levels in their urine samples. This signifies a potential association between heavy metal exposure and CKD. The serum levels of Cu were much higher than expected for CKD patients than controls, and the Zn values were in accordance with established literature. However, the level of Zn in blood was significantly lower in the CKD group compared to the control. This data suggests that the Cu imbalance in the serum of the CKD subjects might have been related to a myriad of reasons, the most plausible of which being exposed to large concentrations of the nephrotoxic metals such as Pb, Cd and Cr in this study. Our study has shed a much needed light on the correlation between CKD and exposure to heavy metals and imbalance of essential metals in blood serum, in a rural locality of Bangladesh.

## 1 Introduction

Bangladesh mainly has an agro-based economy with limited land to feed up a large population. Therefore, the application of pesticides and chemical fertilizers are apparent. A line of evidence suggests potential health and environmental hazards due to pesticide application in Bangladesh, and unregistered pesticide traces in environmental samples also indicate the weakness in the national pesticide's governance (Shammi et al., 2017). Moreover, it has been reported that toxic metals in fertilizers and pesticides might attribute to Chronic Kidney Damage (CKD) (Jayasumana et al., 2015). In Bangladesh, CKD cases have increased at an alarming rate over the past few decades (Anand et al., 2014) (). CKD is a progressive disease that can be identified by a diminished estimated glomerular filtration (eGFR) rate (<60 mL/min/1.73m^2^) that persists for a period of a minimum of three months (Wu et al., 2019). The major known causes of CKD are diabetes, hypertension, hyperlipidemia, structural diseases. Although some studies reveal a possible correlation of CKD with heavy metals, it has not been established for low-level environmental exposure (Jayasumana et al., 2015). Thus, the CKD of unknown etiology is termed as CKDu, which has been considered a matter of choice for environmental health research and nephrological research. The deleterious effects of heavy metals such as Lead (Pb), Chromium (Cr), Cadmium (Cd), arsenic (As), mercury (Hg), etc. on the human body has been documented for some time. However, the correlation of metals and CKD incidence still needs to be explored as minimal research has been done and presented very mixed findings to explain the CKD etiology. For instance, Kim et al. (2015) reported in an epidemiological study that Pb, Hg, and Cd in blood were not associated with CKD, while Cd is associated in CKD cases having comorbidities such as hypertension or diabetes (Kim et al., 2015). In comparison, Chung et al. reported a significant association of blood Pb and Cd with renal dysfunction in Korean adults (Chung et al., 2014). However, a systematic review by Moody et al. summarized a mixed outcome based on epidemiological analysis regarding the association of As-CKD, Pb-CKD, and Cd-CKD. The descriptive studies found elevated Cd and Pb levels in CKD populations (Moody et al., 2018).

The principal organ targeted by Cd is the kidney. CKD has a significant association with elevated blood Cd levels, but less so with blood Pb and Hg (Kim et al., 2015). Though Cd is one of the main factors for Chronic Renal Failure, according to Mohiuddin et al.*,* in the context of Bangladesh, Cr and Pb are much above permissible in fertilizers than, Cd which is almost always below the detection limit (Mohiuddin et al., 2017). Furthermore, in the case of people who have a history of smoking (in stacked years), cadmium accumulation is found proportional with the higher number of years (Lewis et al., 1972).

Zinc (Zn) and Copper (Cu) are essential metals. Alterations in Zinc and Copper metabolism have been frequently observed in patients with chronic kidney disease (CKD) and those with diabetes. These elements have essential roles in the biological systems, as components of proteins, enzymes, and antioxidants. Anorexia, low taste sensibility, hypogeusia glucose intolerance, healing difficulties, and anemia are conventional features of both CKD and diabetes associated with copper and zinc abnormalities. Scarce information exists on the effects of the association between CKD and diabetes in the metabolism of Zn and Cu (Arredondo et al., 2006).

It has been reported that fertilizers contain significant levels of heavy metals with potential nephrotoxic properties (Mohiuddin et al., 2017), which may ultimately result in loss of kidney functions (Barbier et al., 2005; Felley-Bosco and Diezi, 1987; Kim et al., 2015). Jayatilake et al., have found evidence of more significant inhibition of acetylcholinesterase among patients with chronic renal dysfunction in areas of a high prevalence of CKDu (Jayatilake et al., 2013). In their thorough study, they have shown higher numbers of CKDu affected people in the region with high levels of environmental Cadmium (Cd), Lead (Pb), aluminum (Al), and fluoride (F). The commonly used fertilizers in Bangladesh are urea, triple superphosphate (TSP), murate of potash (MoP), diammonium phosphate (DAP), gypsum, magnesium sulfate, etc. A study determined that heavy metal concentration in fertilizers collected from various parts of Bangladesh (Mymensingh, Chattagram, Rajshahi, and Bogra) showed Pb and Cr levels ranging 71.4–168.5 μg g^−1^ and 260–302 μg g^−1^ respectively, in TSP. For DAP it was 21.43–371.4 μg g^−1^ for Pb and 1210–1390 μg g^−1^ for Cr. For MoP the content was 148.6–188.6 μg g^−1^ for Pb and 296–310 μg g^−1^ for Cr. For all the cases maximum allowable limit was 100 μg g^−1^ for Pb, 500 μg g^−1^ for Cr, and 10 μg g^−1^ for Cadmium (Cd) (Mohiuddin et al., 2017). Another study done in several districts of Bangladesh (Faridpur (Sadar), Saltha, Gazipur Sadar, Mymensingh (Sadar), Bagha, Nawabganj (Sadar), Pabna, Baghmara, Gomostapur, Charghat) showed that daily intake of heavy metal of an adult male from those areas is 19.7 μg of Cd, 74.1 μg of Pb, 423 μg of Cr on average (Jahiruddin et al., 2017).

Farmers, in general, belong to the low-income groups and are not well aware of their health risks from heavy metals or concerns for heavy metal or pesticide accumulation in the soil. Their chief objective is to maximize crop yield and, consequently, make a profit (Shammi et al., 2020). A World Bank survey found farmers usually sprayed their crops bare-footed (1% wore sandals), 2% wore gloves, 3% wore protective eye-glasses, and 6% wore home-made cotton masks. Rice is the main staple food and the prevailing harvest in Bangladesh, occupying nearly 80% of the total cropped area. Rice is cultivated through out the year; the crop grown during April to July is known as Aus, rice grown during July to December is called Aman, and that grown from November to May is known as Boro. Moreover, the concerning overuse of fertilizers is lent credibility by the following study conducted in the agrarian area of Tangail. The study showed that 45% of farmers used 201–250 kg of urea per hector for cultivating Aus paddy, 45% of farmers used 201–250 kg of urea per hector for cultivating Aman paddy, 45% of farmers used 201–250 kg of urea per hector for cultivating Boro paddywhere government standard amount was 141, 166 and 269 kg/hector for Aus, Aman, and Boro (varieties of paddy) respectively (Chakrabarty et al., 2014). Therefore, the risk of toxic exposure is high among this occupational group in Bangladesh.

Heavy metals hamper homeostatic metabolic processes by binding to proteins or cation or sulfate/bicarbonate transporters or replacing metals in enzymes or by inducing reactive oxygen species (ROS). Moreover, it is to be noted that these heavy metals are ultimately transported to the kidney for excretion. It happens so that the heavy metals concentrations in the blood are not altered as significantly as in urine. Hence for the study of chronic renal failure due to low-level environmental exposure of heavy metals, it might be a matter of interest to study heavy metals (Pb, Cd, Cr) concentrations in the urine of CKD patients, and also by studying the levels of essential metals (Cu, Zn) in the blood serum to get an intriguingclue about the kidney functions. Therefore, this research aimed to investigate the concentrations of Cd, Pb, Cr in urine and Cu and Zn in the blood serum samples of CKD affected people as well as healthy people. Besides, to examine the possible association between environmental heavy metal exposures and CKD for the first time in Bangladesh.

## 2 Materials and method

### 2.1 Study area and population

A Cross-sectional study on a rural area, namely Sonargaon, Narayanganj, Dhaka, was performed because this area is abundant with industrial and agricultural activities, and geographical means of heavy metal contamination. Narayanganj is flanked by the rivers Shitalaksya and the Buriganga (Banglapedia). Rivers are vital to an agricultural and industrial center. Dumping of industrial effluents, excess fertilizer use, continued irrigation from the rivers have lead to an ecosystem rich in toxic heavy metals.It has been described that the river water, sediment, and fish from the Buriganga and Shitalakshya are highly contaminated with Cadmium, Lead, Chromium, and Arsenic (Islam et al., 2018). Hence these effects in tandem lead to large-scale contamination of the soil, groundwater, crops and affect the general population. Moreover, this area is at a communicable distance from Dhaka city, and there have been many research activities in this area. As a result, people are aware and are more inclined to participate in research. In Sonargaon, this study is the first of its kind.

**Figure 1:**
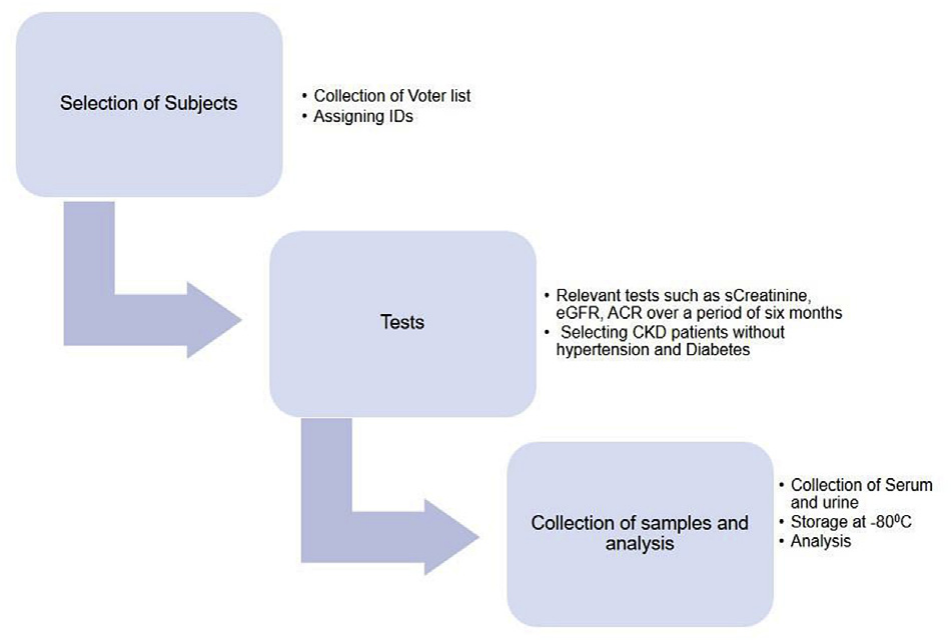
Schematic diagram of our research.

The sampling population selected were adults who were full-time or part-time involved in agricultural activities. A voter list of the 47 villages of Sonargaon was collected, and certain people were requested to appear at the Research sites as volunteers for this study. The selected people were invited to the research center and briefed about the cause of the invitation and were assigned IDs. Researchers along with physicians filled up datasheets, which recorded the healthy and CKD, affected people of the selected area age, profession, habits, occupation, education, monthly income, food habits, etc. The healthy and CKD affected people of the selected area were told to come early in the morning on a different day when their blood and urine were collected. The biological samples (Blood and Urine) of 50 representative people from 16 of the 47 villages were collected in metal-free containers. The controls and CKD affected people of the selected area have been divided into two groups, namely 'exposed' and 'controls'. Structural or functional abnormalities of the kidneys for more than three months as manifested by kidney damage, with or without decreased GFR. In this study, the patients of CKD were confirmed by examining parameters like eGFR, serum creatinine, and ACR over 3 months. Only the confirmed cases of CKD, who did not have High Blood Pressure or Diabetes but had abnormal levels of eGFR and ACR were considered for our study. The controls are the people who have not been diagnosed with CKD (see Figure 1).

This study was approved by the ethics committee of the collaborating hospital and the Institutional Review Board (IRB), Atomic Energy Centre, Bangladesh Atomic Energy Commission, Dhaka, and written informed consent was obtained from all of the volunteers, including patients and healthy controls.

### 2.2 Chemicals and reagents

In our study, all chemical reagents were of analytical grade or supra pure quality (E. Merck, Germany). High purity deionized water (Milli-Q System, Millipore, Thermo scientific, MA USA) with the resistivity of 18.2MΩ cm−^1^ was used for the preparation of all solutions. Nitric acid (HNO_3_) (70%, purified by redistillation, ≥99.999% trace metals basis) was obtained from Sigma Aldrich (St. Louis, MO, USA). Single element standard reference metal solution of Pb, Cd, Cr, Cu, Zn (1000 μg/L; Spectropure, USA) from Agilent Technologies (Santa Clara, CA, USA) was procured. Quality control standard was prepared from multielement standard solution (500 μg/L; Agiland, Santa Clara, USA).

The microcentrifuge tubes (Eppendorf type; Brinkmann Instruments, Inc., Westbury, NY 11590) and pipette tips were unused. They were directly soaked in 5% nitric acid for 24 h, followed by washing in deionized water for another 24 h, and then dried and kept in cleaned capped plastic containers.

### 2.3 Sample preparation and analysis

The urine was transported from the sample collection site in sterilized containers in iceboxes and was finally preserved at -80 °C. Blood was collected in metal-free polypropylene tubes and stored in an icebox. Later it was subjected to centrifugation at 3000 rpm for 15 min at room temperature. Thus, serum was obtained, and the serum was preserved at -80 °C in metal-free polypropylene tubes.

After receiving the serum and urine samples in the Analytical Chemistry Laboratory, dilution of samples was rendered to 10 mL using ultrapure deionized water if required. Analytical calibration standards, internal quality control, and spiked samples with a matrix from known standards were prepared from the standard 1000 ppm stock solution purchased from Spectropure, USA. A calibration curve for each metal of 0.1–10 μg/L was constructed. The Pb, Cr, and Cd in urine and Cu, Zn in serum were determined by Atomic Absorption Spectrophotometer (Varian AA240 FS) equipped with hollow cathode lamps. The wavelength and lamp current of Pb, Cd, Cr, Cu, Zn are 217.0 nm and 10mA, 228.8 nm and 4mA, 357.9 nm and 7mA, 324.8 nm and 4mA, 213.9 nm and 3mA, respectively. The type of flame was Air/Acetylene, the flow rate of Air and Acetylene was 13.50 L/min and 2.90 L/min, respectively. Moreover, the purity of Acetylene gas was 99.99% pure used for the flame. Replicate samples/standards, quality control standard, spike recovery, and method blanks were used to monitor the performance of the instrument and the quality of the data. The recoveries of spiked samples were from 88 to 99%.

### 2.4 Statistical analysis

For statistical analysis SPSS Statistics 22.0 (IBM Corp, Armonk, NY, USA) was used, including analysis of variance, correlation analysis, and principal component analysis. Origin 2019 (OriginLab Corp, Northampton, MA, USA) was used for distribution tests and charting. The Monto Carlo simulation was performed using Crystal Ball Software (11.1 Oracle Inc., Oracle, CA, USA).

### 2.5 Monte Carlo simulation

**Figure 2:**
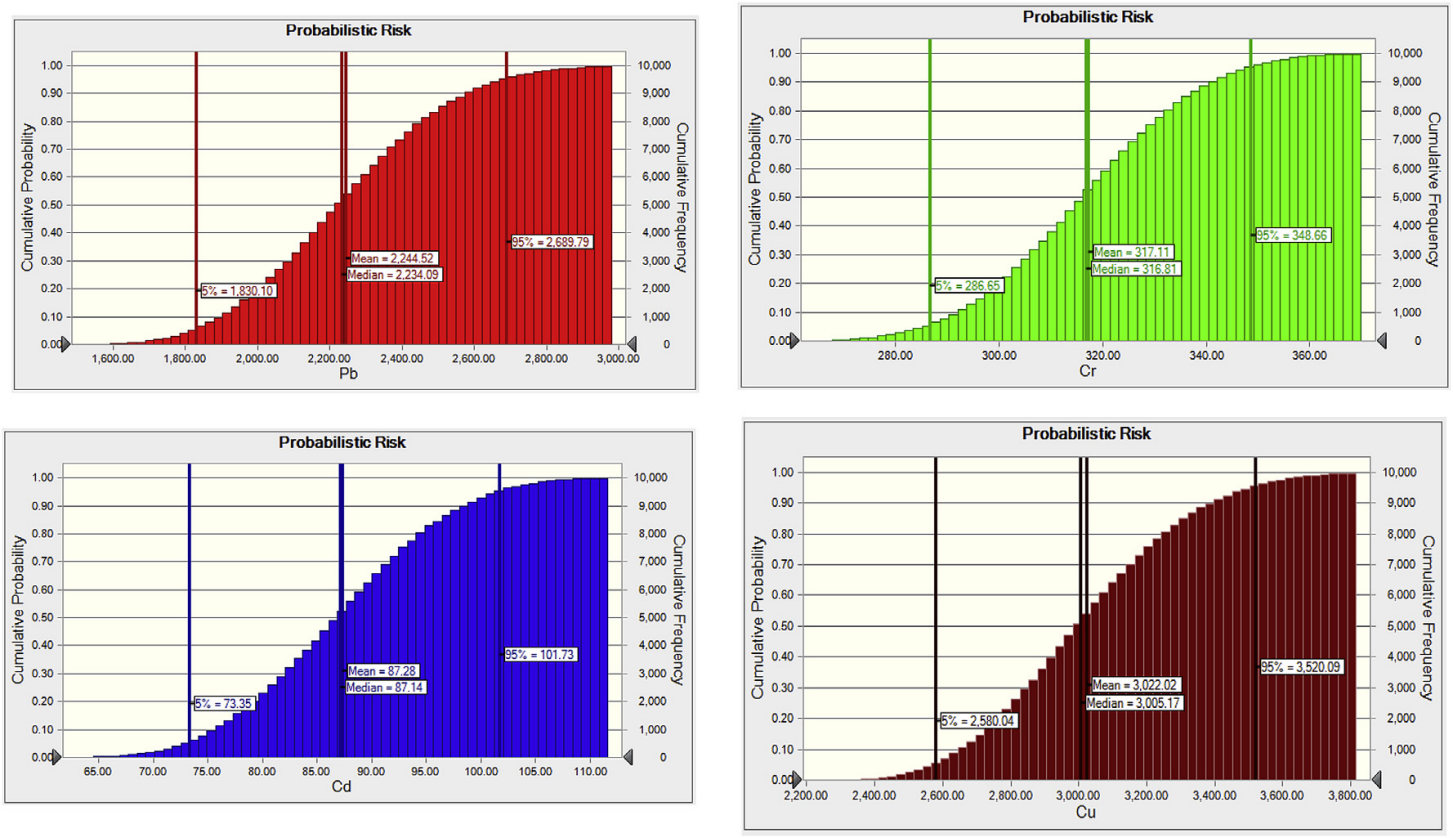
Cumulative distribution of Pb, Cd, Cr, Cu for probabilistic risk of CKD patients and controls in study area.

The probabilistic risk assessment of heavy metals was performed using a simulation of Monte Carlo. Weak correlation or independence between the input variables was assumed in this simulation. Input variables (Pb, Cd, Cr, Cu) were modeled as specific probability distribution functions (Table 1). To ensure the reliability of the results, 20,000 random iteractions of each input variable were carried out in each simulation. The input variables were randomly extracted from the defined probability distributions (Table 1). In this study, the 5th, 25th, 50th, 75th, and 95th percentiles for each heavy metal were extracted from the probability distribution (Figure 2).

**Table 1:** The probability risk distribution of heavy metals in patient.

Probability	Pb	Cd	Cr	Cu	Zn
5%	1830.10	73.35	286.65	2580.04	1922.46
50%	2234.07	87.14	316.81	3005.15	2242.86
75%	2420.60	93.25	329.96	3210.46	2388.39
95%	2689.79	101.73	348.66	3520.09	2604.84

## 3 Results

### 3.1 Metals concentration levels in the urine samples

**Figure 3:**
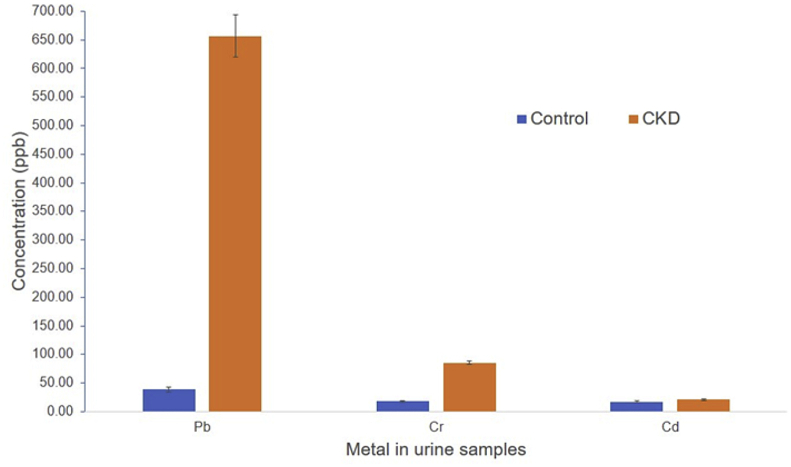
Comparative average concentrations of metals (±SD, n = 35) in the urine of CKD patients and controls. Asterisk (∗) denotes a significant difference at p < 0.05 compared to the control.

The concentrations of metals in the urine samples of CKD patients and control subjects with their descriptive statistics are presented in Table 2. Pb levels in urine samples from CKD patients are ranging from 30 μg/L to 1040 μg/L with a mean value of 594.86 μg/L. In the control group, the mean value of Pb was 38.48 μg/L (ranging from 10 to 90 μg/L) (Table 2). In the case of Cr in the urine sample, the mean concentration of 77.38 μg/L was found for CKD having a range from 2.75 to 116 μg/L. On the other hand, in the control group, the mean concentration of Cr was 18.00 μg/L with a range of 5.6–29.09 μg/L (Table 2). The mean concentration of Cd in the urine samples of CKD cases was 19.69 μg/L with a range from 1.56 to 45 μg/L. Whereas, mean Cd concentration of 17.50 μg/L was found in the control group with a range from 8 to 30 μg/L in the urine samples (Table 2). Comparative status of Pb, Cr, and Cd in urine samples from CKD cases and healthy counterpart is presented in Figure 3. For Pb and Cr, a significantly elevated concentration was found in the urine samples of CKD patients compared to healthy people. However, in the case of Cd, the difference in concentration level was not significant between CKD and healthy people (Figure 3).

**Table 2:** Descriptive statistics for the metal's concentrations in urine and blood samples of CKD cases (n = 35) and control cases (n = 20).

	N	Minimum	Maximum	Mean	Std. Deviation	Variance	Skewness		Kurtosis	
	Statistic	Statistic	Statistic	Statistic	Statistic	Statistic	Statistic	Std. Error	Statistic	Std. Error
CKD urine Pb	35	30	1040	594.8603	256.0849	65579.45	-0.395	0.398	-0.057	0.778
CKD urine Cr	35	2.75	116	77.3883	26.64065	709.724	-1.402	0.398	1.745	0.778
CKD urine Cd	35	1.56	45	19.6937	9.10494	82.9	0.895	0.398	1.532	0.778
CKD blood Cu	35	30	1185.31	737.0878	298.1705	88905.65	-0.779	0.398	0.422	0.778
CKD blood Zn	35	29	1042.31	519.7827	222.1036	49330.01	-0.048	0.398	0.867	0.778
Control urine Pb	20	10	90	38.4825	22.463	504.587	1.14	0.512	1.045	0.992
Control urine Cr	20	5.6	29.09	18.0025	7.87408	62.001	0.065	0.512	-1.51	0.992
Control urine Cd	20	8	30	17.507	7.3631	54.215	0.108	0.512	-1.18	0.992
Control blood Cu	20	214.45	690.89	511.5831	127.9366	16367.79	-0.694	0.512	-0.04	0.992
Control blood Zn	20	636.78	1054	771.8009	113.3127	12839.76	0.968	0.512	0.458	0.992

### 3.2 Metals concentration levels in the blood samples

**Figure 4:**
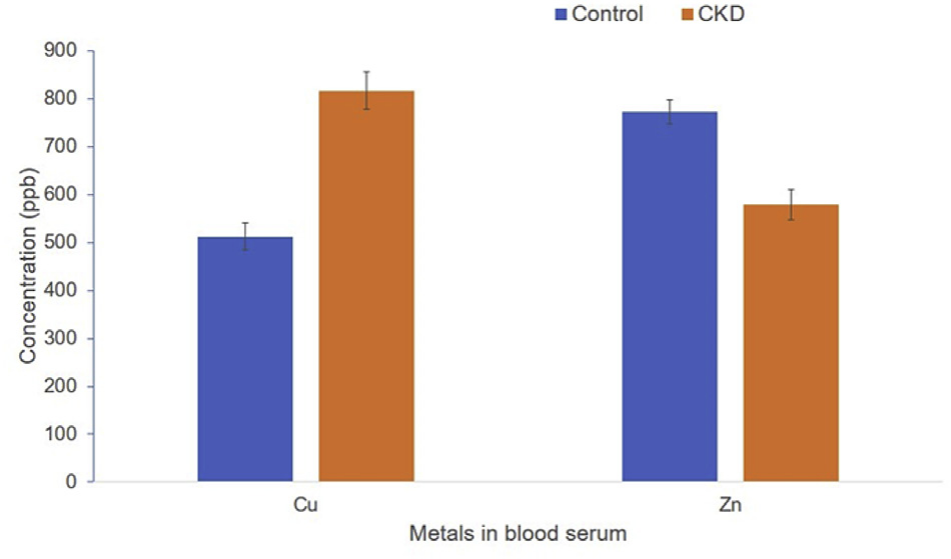
Comparative average concentrations of metals (±SD, n = 20) in the blood serum of CKD patients and controls. Asterisk (∗) denotes a significant difference at p < 0.05 compared to the control.

In the blood samples of CKD patients and their healthy counterparts, two metals were studied, i.e., Cu and Zn. The descriptive statistics of the data are given in (Table 2). The mean concentration of Cu in blood samples was 737.08 μg/L in the CKD group ranging from 30 μg/L to 1185.31 μg/L. In the case of Zn, the mean concentration in blood samples of CKD cases was 519.78 μg/L having a range from 29 to 1042.31 μg/L (Table 2). On the other hand, for the healthy control group mean Cu concentration in blood samples was 511.58 μg/L with a range of 2014.45 to 690.89 μg/L. In contrast, Zn in blood samples of the control group ranged from 636.78 μg/L to 1054 μg/L with a mean of 771.8 μg/L (Table 2). Statistical comparison was conducted to examine the differences between these two metals in the blood of both study subjects and results are presented in Figure 4. For both of the metals, a significant difference was found. Cu is significantly increased in the blood samples of CKD cases compared to their healthy counterpart (Figure 4) 3. On the other hand, Zn was found significantly lower in the CKD group compared to their healthy control group (Figure 4).

### 3.3 Pearsons' correlation and PCA of the metals in the study groups

Pearsons' correlation was performed to find a possible association among different variables (Table 3). A significant positive correlation was found between Pb concentration in CKD patients' urine sample and urine level Cr and blood level Zn in CKD group at p < 0.01, and with urine level Cd, blood level Cu at p < 0.05 (Table 3). Urine level of Cr in the CKD group also showed a significant association with urine level Pb, blood level Cu, and Zn in CKD patients. However, urine level Cd was only significantly associated with the blood level of Zn in CKD cases at p < 0.05 (Table 3). In addition, the Blood level of Cu in CKD cases is significantly associated with the blood level of Zn in the same group. Moreover, the Pb in urine level was significantly associated with the urine level Cd in the control group. Four principal components were identified with significant association PC1 comprised with a strong influence of urine level Pb and Cr, and Cd in CKD group followed by PC2 formed with urine level Pb and Cd in the control group; PC3 comprised of blood level Cu and Zn in CKD, and negative association of blood level Cu in the control group; and PC4 made of urine level Cr and blood level Zn in the control group (Table 4).

**Table 3:** Pearson correlation for among different variables in the study.

	P_Pb_U	P_Cr_U	P_Cd_U	P_Cu_B	P_Zn_B	C_Pb_U	C_Cr_U	C_Cd_U	C_Cu_B	C_Zn_B
P_Pb_U	1									
P_Cr_U	.776∗∗	1								
P_Cd_U	.401∗	.396∗	1							
P_Cu_B	.405∗	.675∗∗	0.168	1						
P_Zn_B	.551∗∗	.542∗∗	.368∗	.614∗∗	1					
C_Pb_U	-0.041	0.001	-0.301	0.013	-0.219	1				
C_Cr_U	0.239	0.016	-0.124	0.013	-0.058	0.365	1			
C_Cd_U	-0.092	-0.047	-0.251	0.148	-0.342	.462∗	0.079	1		
C_Cu_B	-0.151	-0.223	-0.282	-0.172	-0.436	-0.009	-0.02	0.158	1	
C_Zn_B	-0.095	-0.296	-0.385	-0.01	-0.209	0.042	0.204	0.057	0.108	1

P: CKD; C: Control; U: Urine; B: Blood Serum.

∗ Correlation is significant at the 0.05 level (2-tailed).

∗∗ Correlation is significant at the 0.01 level (2-tailed).

**Table 4:** Rotated component matrix for principal component analysis (PCA) analysis.

Parameters	Component 1	Component 2	Component 3	Component 4
	PC1	PC2	PC3	PC4
P_Pb_U	**0.88**	-0.103	0.069	0.197
P_Cr_U	**0.86**	0.113	0.011	-0.155
P_Cd_U	**0.578**	-0.311	0.283	-0.36
P_Cu_B	-0.34	0.353	**0.55**	-0.196
P_Zn_B	0.124	-0.473	**0.658**	0.01
C_Pb_U	0.045	**0.752**	0.039	0.336
C_Cr_U	0.2	0.256	0.127	**0.77**
C_Cd_U	-0.069	**0.811**	-0.139	-0.051
C_Cu_B	-0.184	0.061	**-0.806**	-0.029
C_Zn_B	-0.349	-0.089	-0.188	**0.679**
Eigenvalues	2.181	1.77	1.541	1.402
% of Variance	21.812	17.698	15.411	14.015
Cumulative %	21.812	39.51	54.921	68.936

### 3.4 Probabilistic health risk assessment

Considering the variation of concentrations of heavy metals in urine and serum samples, risks were simulated using the Monte Carlo method. The 5th, 25th, 50th, 75th, and 95th percentile values of the distributions of heavy metals are shown in Figure 4. The Pb, Cu were higher than Cr, Cd one even at the 5th percentile, suggesting that most of the residents were exposed to a significant health risk for Pb, Cu. The order of the 95^th^ percentile risk value of metals studied in the control and CKD group showed the pattern as follows: Cu > Pb > Cr > Cd (Figure 2). A similar trend was observed in the case of 75^th^, 50^th^ and 5^th^ percentile.

## 4 Discussion

In this study, it was found that the concentration of serum Copper lies within 214.45–1185.31 μg/L and serum Zinc within 319.45–1042.31 μg/L. DiDonato et al.(DiDonato and Sarkar, 1997) reported that serum Copper for healthy human beings must lie between 100 and 150 μg/L and Barsoum et al. (Barsoum, 2006) reported that serum Zinc must lie between 660-1100 μg/L. It appears that the mean values obtained for serum Copper are much more than their recommended values, while the mean of the serum Zinc levels are within the recommended limit. However, the mean for Copper is less than the Zinc values as per the recommendations of DiDonato and Barsoum. The mean for serum Copper for case and control are 816.99 μg/L and 511.49 μg/L, respectively. The increase in the copper levels in CKD affected people are expected (Shih et al., 2012). The mean serum Zinc level for the case and control are 578.35 μg/L and 771.80 μg/L, respectively. The decrease in levels of zinc found in this investigation is also expected as per Shih et al. (2012). The mean serum Copper and Zinc for cases were higher and lower, respectively than that of controls, which is in agreement with the literature. However, it must be noted that the mean for copper concentration in serum found is about four times higher than 150 μg/L (the recommended value). At this point it might be concluded that this has resulted from a copper-rich diet of the people of Sonargaon and a chronic long term exposure to heavy metals. Also, the standard deviations were quite large indicating that both serum Copper and Zinc data varied greatly from man to man.

Out of 50 urine samples, 30 of them have a very high concentration of Pb (250–1040 μg/L), and only 20 samples have Lead concentration levels (10–90 μg/L), which are comparatively lower. According to Yaman et al. (Yaman, 1999), the normal levels of Lead in the urine may be assumed to center about a mean of 35 μg/L. For unexposed human urine, the Lead and Cadmium concentration must be below 80 μg/L and 1 μg/L, respectively. The mean urinary Lead for cases and controls are 656.50 μg/L and 38.48 μg/L. It is evident that the CKD patients have much higher levels of Lead in their urine in contrast to controls. The highest urinary Lead excretion mean values are obtained for individuals from villages named Panam Gabtoli, Uttar Khansardi, and Hariya Chowdhury para, which are 1040, 926.67, and 910 μg/L, respectively. And the lowest values are obtained for individuals from villages named Bhabanipur, Khamargaon, Dighi Chandpur, Dakarband, and H. Khaser Kanda, which are 10, 20, 30, 35, and 55 μg/L, respectively. The urinary excretion of Lead is very high in people of 10 of the 16 villages, which is much more above the normal range. It might be possible that the former areas are much more contaminated with Lead than the latter due to heavy exposure to industrial effluents containing Lead or by overuse of fertilizers containing Lead.

The mean urinary Cadmium for case and control was 20.67 μg/L and 17.51 μg/L, respectively. The CKD patients had higher levels of Cadmium in their urine in contrast to controls. This agrees with the reported trend (Barbier et al., 2005) but to a limited extent. The highest mean values for urinary Cadmium excretion were found for individuals from locality Uttar Khansardi, Panchabati, Hariganj, Basundardi, H, Khaser Kanda, Bhabanipur, Dhanpur, Panam Gabtoli and Haria Chowdhury para which was 45.00, 30.00, 23.00, 22.00 and 21.00 μg/L respectively. The lowest values were obtained for individuals from localities Dighi Chandpur, Khamargaon, Haria Khaser Kanda, and Paschim Damodari, which were 8.00, 8.00, 8.00, 9.90 μg/L, respectively. It might be possible that the former areas are much more contaminated with Cadmium than later. However, no area is under the safe limit of healthy levels of urinary excretion of Cadmium. This finding is very alarming as we know Cadmium is a known carcinogen and a toxic metal.

The mean urinary Chromium, for exposure and control, are 85.35 μg/L and 18.00 μg/L. Kiilunen et al. (1987) have found the average value for urinary excretion of Cr for normal or unexposed human beings is 0.125 μg/L and recommended that the values must be below 0.57 μg/L. It is evident that the CKD patients had higher levels of Chromium in their urine in contrast to controls. All members from the villages had urinary chromium excretion values that were very high, except individuals from localities Dakarband, Vhabanipur, and Dakarband, which had means of 8.00, 5.60, and 12.00 μg/L, respectively. It might be possible that all the areas under study are contaminated with Chromium. However, no area was under the safe limit of healthy urinary excretion of Chromium.

Though the urinary excretion of Cadmium and Chromium are lower in the 16 villages, they are well above the recommended range for almost all of the villages. The urinary excretion of all three metals is highest for Panam Gabtoli and Haria Baidyer Para, while the lowest values can be attributed to Bhabanipur, Khamargaon, Dighi Chandpur, and Dakarband.

The above paragraphs depict that the urinary excretion of Lead, Cadmium, and Chromium are higher for the cases than the controls. One of our principle research parameters is that the CKD patients who did not have hypertension or diabetes were specifically chosen. It proves a positive correlation between chronic exposure to nephrotoxic heavy metals and CKD. Also, this exposure might have caused an imbalance of Copper concentration in their blood serum, which have affected their kidneys and might affect other internal organs in the long term. These findings also support the out of the probalilistic health risk assessment of this study (Figure 2). The highest risk was observed due to high exposure of Cu followed by the Pb. Accumulation of Cu in the serum of CKD indicates the lower urinary excretion of Cu might be associated with the high probalistic risk factor for CKD patients in this study. This may also be concluded that CKD patient might be affected by low serum Zn level which triggers the low profile of antioxidants in the patient might be stimulated by the high level of Cu. However, these findings warrant further large scale study in this issue to clarify the exact mechanism.

## 5 Conclusion

In comparison with other lethal ailments, CKD is comparatively obscure in Bangladesh. This collaborative study being the first of its kind in Bangladesh, has established a positive correlation between CKD and chronic exposure to heavy metals. Which is essentially supported by the fact that the residents with the highest values of Pb, Cd, Cr in their urine suffered from CKD and an imbalance of Cu concentrations in their blood serum. Although in our study, a positive correlation was established, further research into the correlation between nephrotoxic metals and CKD is necessary to comprehend CKDu and its treatment.

## Declarations

### Author contribution statement

Tasrina Rabia Choudhury: Conceived and designed the experiments; Performed the experiments; Analyzed and interpreted the data; Contributed reagents, materials, analysis tools or data; Wrote the paper.

Sk. Zubaer Zaman: Analyzed and interpreted the data; Wrote the paper.

Tanzina Iveen Chowdhury & Md. Anwarul Islam: Conceived and designed the experiments.

Bilkis Ara Begum: Contributed reagents, materials, analysis tools.

Md. Mostafizur Rahman: Analyzed and interpreted the data.

### Funding statement

This work was supported by 10.13039/100007225Ministry of Science and Technology, Government of the People’s Republic of Bangladesh (Grant No. 39.00.0000.012.002.04. 19-06, merit 193, serial 229).

### Data availability statement

Data included in article/supplementary material/referenced in article.

### Declaration of interests statement

The authors declare no conflict of interest.

### Additional information

No additional information is available for this paper.
